# The Triple Axis and SPINS Spectrometers

**DOI:** 10.6028/jres.098.005

**Published:** 1993

**Authors:** S. F. Trevino

**Affiliations:** ARDEC Picatinny Arsenal, NJ 07806; National Institute of Standards and Technology, Gaithersburg, MD 20899

**Keywords:** condensed matter spectroscopy, dispersion curves, hydrogen vibrations and translational diffusion, inelastic neutron scattering, magnetic excitations, neutron spectrometer, physical chemistry spectroscopy, polarized neutrons, rotational diffusion, SPINS spectrometer, solid state tunneling, triple axis spectrometer

## Abstract

In this paper are described the triple axis and spin polarized inelastic neutron scattering (SPINS) spectrometers which are installed at the NIST Cold Neutron Research Facility (CNRF). The general principle of operation of these two instruments is described in sufficient detail to allow the reader to make an informed decision as to their usefulness for his needs. However, it is the intention of the staff at the CNRF to provide the expert resources for their efficient use in any given situation. Thus, this work is not intended as a user manual but rather as a guide into the range of applicability of the two instruments.

## 1. Introduction

The triple axis spectrometer is the most widely used instrument in the study of materials with neutron scattering. No steady state source of neutrons (nuclear reactors) intended for use as a research tool with neutron scattering can be said to be complete without at least one such instrument installed. The concept and initial construction of this type of instrument is due to B. N. Brockhouse [1] who in the early 1950s installed the first model on the reactor located at Chalk River, Canada. This instrument was used to determine in detail for the first time the phonon properties of many different types of simple materials. Its control was primitive compared to that available today with the technology of robotics. Many improvements and expanded capabilities have been incorporated since the first prototype, producing a versatile instrument which has been used in the study of a wide variety of materials. The instrument was intended for the study of elementary excitations in condensed matter. Changes in the energy and momentum of the neutrons upon scattering by the sample are measured in a straight forward manner. These changes are due to the interaction of the neutron with the excitations which are supported by the sample under investigation and constitute a direct measure of the character of the excitations.

The CNRF instruments provide moderate resolution (0.01–1.0 meV) with sufficient intensity for use in a wide range of problems. They are ideally suited for the study of phonon dispersion curves in single crystals, tunneling modes of energies greater than ≈0.025 meV, quasielastic scattering studies of rotational and nonlocal diffusion in the time regime of picoseconds, vibrations of surfaces or molecules adsorbed on surfaces and phonon density of states for that large class of materials which contain hydrogen. Specific mention of the applicability of neutron scattering to the study of hydrogeneous materials should be emphasized here. The hydrogen nucleus has the largest cross section (scattering interaction) for neutron scattering and is predominantly incoherent. Hydrogen vibrations have been detected in samples containing as little as 0.01 mol total hydrogen in the sample. Because the instrument is energy sensitive, it can also be used to measure purely elastic scattering whether it be due to coherent (nuclear or magnetic) or incoherent events. Information on the time-averaged structure of the atomic and molecular constituents of the sample is therefore accessible. Finally, the ability of producing and analyzing polarized neutrons allows more detailed measurement of the magnetic properties of the sample. These magnetic properties can be static, i.e., a structural description of the magnetic moments, or dynamic such as magnons.

The range of energies (0.025–14 meV) of excitations accessible to these instruments is substantially larger (although with poorer resolution) than available with the spin-echo and backscatter spectrometers. Independent control of the momentum (*Q*) and energy transfer (*E*) is routine if required as opposed to the time of flight spectrometer in which *Q* and *E* are related by the instrumental configuration.

The theory of operation, including considerations such as the factors which determine the resolution, various neutron filters available, and other innovations which enhance the usefulness of the instrument will be described in the next section. That section will include a description of the two instruments installed on neutron guides at the CNRF. The last section presents results of several measurements with a triple axis spectrometer.

## 2. Fundamentals of the Technique

### 2.1 The Triple Axis Spectrometer

**Fig. 1:**
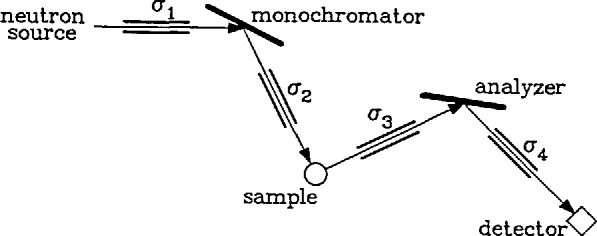
A schematic diagram of the triple axis spectrometer.

In Fig. 1 is exhibited a schematic drawing of a triple axis spectrometer. The derivation of the name “Triple Axis Spectrometer” becomes clear from an inspection of this figure. There are three vertical axes about which parts of the machine rotate. The first, labeled monochromator, allows a narrow band of neutron wavelengths to be chosen from the much broader spectrum which is provided by the neutron source. The spectrum of this band is centered at a wavelength λ defined by Bragg’s law for the diffraction of radiation by a crystal which is:

nλ=2dsin(θ) (1)

where *d* is the lattice spacing of the monochromator crystal, 2*θ* is the angle through which the neutrons are scattered, and *n* is a positive integer (*n* = 1 is the first order, *n* =2 the second, etc.). This angle is defined by the two collimators located on either side of the monochromator crystal which, along with the mosaic angular spread of the monochromator crystal, determine the width (in wavelength) of the spectrum of neutrons exiting from the second collimator and illuminating the sample. The second axis of rotation passes through the sample and allows for the investigation of the neutron scattering properties of the sample as a function of the scattering angle *ϑ.* The third axis passes through the analyzer crystal. The function of this latter part of the instrument is to determine the center and width of the band of neutron wavelengths to which the detector will respond. The principle by which this is accomplished is completely analogous to that used in the case of the monochromator. The band width will be determined here by the angular divergence of the last two collimators and the characteristics of the analyzer.

The magnitude of the wave vector ***k*** of the neutron (or of any radiation which is characterized by a wavelength λ) is defined as

k=2π/λ, (2)

with the direction of the vector ***k*** being that in which the neutron travels. In terms of ***k*** the energy of the neutron is

$$E=\hbar^{2}k^{2}/2m.$$ (3)

It can be seen that the triple axis spectrometer is capable of defining ***k***_0_ (and therefore *E*_0_) of the neutrons incident on the sample, ***k*** (and *E*) of the neutrons scattered by the sample, the wave vector transfer ***Q***

$$Q=k_{0}-k$$ (4)

whose magnitude is, from the law of cosines

$$Q^{2}=k_{0}^{2}+k^{2}-2k_{0}k\cos\vartheta ,$$ (5)

and the energy transfer *ħω*

$$\hbar \omega =E_{0}-E.$$ (6)

The great power of this spectrometer is that it allows choosing arbitrarily these two quantities, ***Q*** and *ħω* (subject to kinematic constraints), in terms of which the most detailed properties of the scattering law of the sample depend. In turn, the properties of the sample which are reflected in the scattering law will be revealed through its determination [2]. The scattering law of the sample could be dependent on its orientation relative to the wave vector transfer ***Q***. This is certainly true in the case in which the sample consists of a single crystal. Other examples include one dimensional orientation of polymers and two dimensional order produced by epitaxial growth. The instrument is capable of independently producing any desired relative orientation of sample and ***Q***.

### 2.2 Resolution

The resolution of the instrument will determine its utility for a given measurement. A general rule with this instrument, as with most, is that resolution is purchased at the expense of intensity. This should be kept in mind when configuring the instrument for a given measurement. The parameters which govern the resolution are the angular divergence of the four collimators (*σ*^1^, *σ*^2^, *σ*^3^, *σ*^4^, see Fig. 1), the mosaic divergence *η*_m_ and *η*_a_ of the monochromator and analyzer crystals, and the Bragg angles *θ*_m_ and *θ*_a_ of the monochromator and analyzer, respectively. The mosaic divergence of a crystal is usually not available for change since it is a physical characteristic of that crystal. A change of this parameter would require a change of the crystal. Most crystals when first grown have a mosaic which is much too perfect (≈ 1 min) to be useful as monochromators. The integrated reflectivity is a function of the mosaic of the crystal, being smaller for smaller mosaic. In general, monochromators are used with mosaic divergence of 15–30 min of are. Techniques for treating virgin crystals to produce such a mosaic have been successfully applied to many different (but not all) crystals including Cu, Si, Zn, and Ge. The value of the divergence of a collimator is determined by the spacing *d* between the vertical blades and the length *l* of the blades (to a very good approximation *σ*=*d/l* rad). It is standard practice to have available several collimators (≈4) varying in values of the divergence from 5–80 min of are for each of the four positions. The scattering angles from the monochromator and analyzer are continuously variable. The value of each is dictated by the lattice spacing of the crystal and the neutron energy required from it [see Eqs. (1–3)]. The relationship of the resolution to these parameters has been well investigated and the results confirmed experimentally [3]. Computer codes which allow the calculation of both the energy and momentum resolution as functions of all the relevant parameters are available for the efficient planning of any given measurement. Typical values of the energy resolution are a few percent of the energy transfer.

A straightforward differentiation of Eq. (1) leads to the expression

**Fig. 2:**
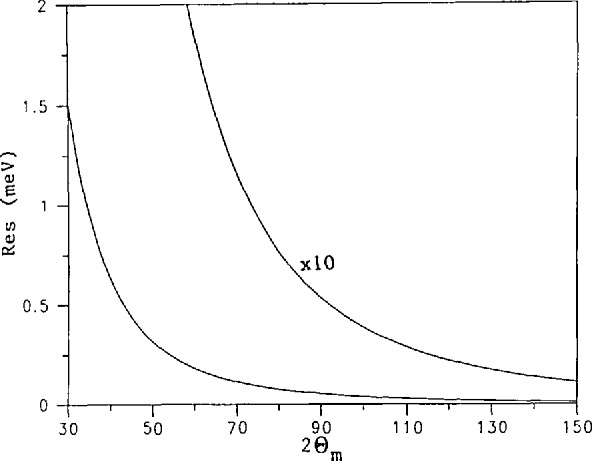
The calculated resolution of a triple axis spectrometer as a function of the monochromator scattering angle 2*θ*_m_. The collimation widths are 40, 20, 20, and 20 min of are for the collimators before and after the monochromator and before and after the analyzer, respectively. The monochromator and analyzer crystals are both PG (002). The resolution is for elastic scattering (*E*=*E*_0_).

Δλ/λ=cotθΔθ, (7)

which relates the effect on the wavelength band width of the scattering angle from both monochromator and detector. A simple method to obtain better resolution would seem to be to increase the monochromator and analyzer scattering angle to as large a value as possible. From Eq. (1), it is seen that this process would produce neutrons of long wavelength (low energy). In order to use this effect productively, there must exist in the spectrum of neutrons incident on the monochromator a sufficient number of low energy (cold) neutrons. Thus the present effort. In Fig. 2 is presented the result of a calculation of the resolution of a triple axis spectrometer as a function of the monochromator scattering angle for the conditions given in the caption. The effect is dramatic.

### 2.3 Filters

Recall from Eq. (1) that a crystal with a given lattice spacing reflects neutrons of several wavelengths, viz. the several orders. The higher order neutrons being of shorter wavelength, viz. *λ*/2, *λ*/3, etc., are of higher energy, 4*E*, 9*E*, etc. It is usual to place a filter either in the beam incident on the sample or in that scattered from the sample in order to reduce the “contamination” of these orders so that a clean measurement is possible. Two of the most widely used filter materials for this application are beryllium (Be) and pyrolitic graphite (PG). The mechanisms by which filtering is produced will not be discussed here but only the resulting properties. Polycrystalline Be is an extremely effective low-pass filter. The cutoff energy is ≈5 meV. The rejection ratio for neutrons of energies larger than the cutoff to those smaller than the cutoff is a function of the length and temperature of the filter. As an example, for a filter of 100 mm length at a temperature of 78 K (liquid nitrogen), the rejection ratio is 3 × 10^5^ with a transmission of the low energy neutrons of 0.95. More effective rejection can be obtained if required by a particular experiment at a small cost of transmitted low energy neutrons by using a longer cooled filter. There is, unfortunately, no universal filter for energies larger than 5 meV. The most useful filter in this energy region is PG for energies of 13.7, 14.8, 28., 30.5, and 40.3 meV. These are energies for which the transmission of the filter is reasonable (≈0.7 for a length of 50 mm) with a rejection of 10^4^ for the second order energies. An alternate method of obtaining a “clean” beam is to use a monochromator whose properties are such that the second order reflection, for example, is forbidden. Several planes of Si, which has the diamond structure, satisfy this requirement. The technology for treating virgin Si crystals, which are usually too perfect (having very small mosaic ≈1 min of are resulting in a very small reflectivity), is only now becoming available. It is always true that care must be exercised to ensure that a measured resonance is due to a property of the sample and not some instrumental effect due to a characteristic of the monochromator or analyzer.

### 2.4 Polarized Neutrons

Because the neutron possess a nuclear magnetic moment, the scattering from a sample which exhibits magnetism will be sensitive to the properties of the sample. The scattering is, of course, still also a function of the nuclear positions and motions. If the resonance produced by the magnetic properties of the sample is well separated from that produced by the nuclear scattering, no further effort is required. If however there is required unambiguous identification of the resonances as arising from a magnetic source, one can increase the sensitivity of the scattering to the magnetic properties, over those which depend only on the position and motions of the nuclei, by using a beam of space polarized neutrons. The scattering law can then be measured as a function of whether the spin of the neutrons is reversed or not in the scattering. Such a spin flip can only be produced by magnetic interactions. This type of measurement is therefore capable of distinguishing that part of the scattering which is due to the magnetic properties of the sample from that which is not. Technologies capable of producing polarized neutrons and effecting their spin flip are required for such measurements.

The production of polarized neutrons has been effected by using magnetic monochromator crystals whose scattering is strongly dependent on the relative orientation of the neutron magnetic moment and a magnetic field (the guide field) extending from the monochromator to the sample (or the sample to the analyzer). The three most widely used materials for this purpose are single crystals of CoFe, Fe^57^, and Heusler alloy. The first two are used for neutrons of wavelengths 0.05–0.15 nm (0.5–1.5 Å) and the last for wavelengths of 0.1–0.4 nm (1–4 Å). For neutrons of wavelengths longer than 0.4 nm (4 Å), such as those available at the cold neutron facility, devices constructed of bilayers, a few nanometers (tens of Å) thick, alternately of a magnetic and non-magnetic material, have been constructed with the desired properties (these devices are known as polarizing super-mirrors). The efficiency of neutron polarization of these devices is on the order of 98 percent or better. The additional sensitivity to magnetic scattering obtained in this manner is of course not without cost in overall sensitivity in that at most 1/2 of the neutrons incident on the polarizing devices will be scattered by them and available for the measurement. The trade off in most cases is however well worth the effort. In addition to the ability to produce and be sensitive to a particular polarization of the neutron, one must be able to effect a rotation of the polarization of the neutron beam either before or after scattering from the sample. This can be accomplished by passing the neutrons through a magnetic field (the flipping field) directed perpendicular to the plane defined by the neutron wavevector ***k*** and the guide field. The mechanism operative in this device is that of matching the Larmor frequency of the neutron in the flipping field to the flight time of the neutron in this field to produce an arbitrary (usually 180°) rotation of the magnetic moment. A second coil is placed between the guide field and the flipping field whose function is to cancel the guide field from the region of space occupied by the flipping field. These devices are well understood and readily available.

### 2.5 Focusing

In the main, the measured quantities of interest are ***k*_0_** and ***k*** and these depend on the vertical collimation only in second order. This allows the use of devices capable of vertical focusing to increase the signal without unduly affecting the resolution. One such device is a monochromator which consists of several crystals mounted so that each can be rotated about an axis parallel to the scattering surface of the crystals. The crystals are arranged so as to approximate a cylindrical scattering surface with the normal to the surface along the scattered neutron direction and with radius of curvature *R.* The relation between the neutron wavelength, the curvature radius, and the distance between neutron source-monochromator and monochromator-sample is obtained from simple optical considerations [4]. Such a device is capable of producing an increase in the neutron flux on the sample by a factor of ≈2.

### 2.6 Spin Polarized Inelastic Neutron Scattering (SPINS)

In 1962 G. M. Drabkin [5] proposed a scheme by which a beam of polarized neutrons could be produced whose energy is determined by the state of a magnetic field through which it passes rather than by the angle through which it is scattered from a crystal. This method is in principle capable of modifying one of the characteristics of a conventional triple-axis spectrometer. In the standard spectrometer, the energy resolution and the momentum (wave vector ***Q***) resolution, both of which are dictated by the characteristics of the crystals and collimations used, are strongly coupled. In many applications, this is not a particular disadvantage in that a well-defined resolution function is very useful. There would in many instances be an advantage in signal if the momentum resolution could be substantially relaxed with respect to the energy resolution by decoupling the two. Examples of such situations include dispersionless optic modes in crystals and single particle vibrations of hydrogen. The method which Drabkin has proposed produces just such a decoupling.

**Fig. 3:**
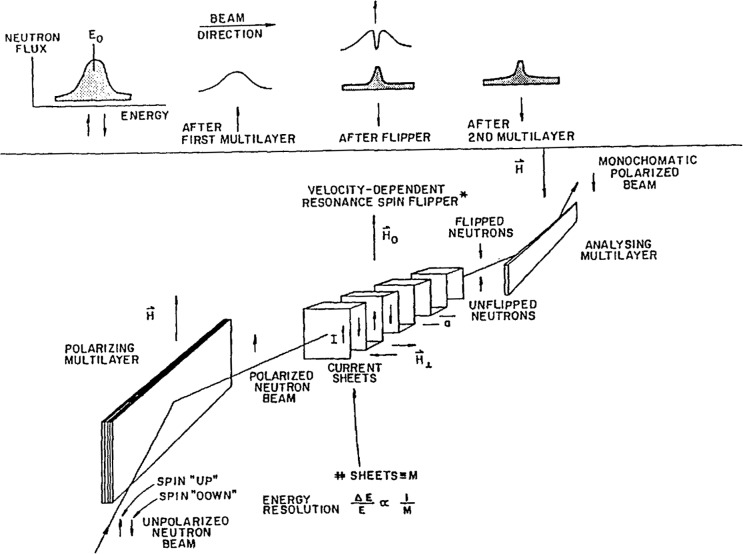
A schematic diagram of the Drabkin neutron spin-flipper device.

The device can be understood by consulting Fig. 3. An unpolarized beam with a relatively broad energy bandwidth is first polarized by a super-mirror. The beam next traverses a current carrying foil which is folded in such a manner as to produce a small, spatially oscillating magnetic field *H*_⊥_ which is normal to both the propagation direction of the beam and to a larger uniform magnetic field *H*_0_ that is superimposed over the full extent of the foil. These two fields are related according to *H*_⊥_ = 2*H*_0_*/M* where “*M*” is the number of spaces between adjacent current sheets through which the neutrons pass. The resultant magnetic field acts as a velocity or energy selective resonance flipper. Only those neutrons with velocities near *υ*_0_ = *a* γ*H*_0_/π (where γ is the gyromagnetic ratio of the neutron and “*a*” is the spacing between adjacent sheets of the foil) will undergo a spin-flip with a high probability. The probability distribution as a function of velocity is centered at velocity *υ*_0_ and has a width proportional to 1/*M*. Those neutrons which are not flipped are subsequently not reflected by a second supermirror in which the magnetization direction is opposite to that of the first. Thus, the pair of supermirrors and the flipper act as an energy-dependent filter whose characteristics are controlled electrically. Table 1 compares the relative intensity to be expected for a given energy resolution obtained with a spin flip analyzer compared to the conventional triple axis configuration. Note that as the energy resolution is increased, the advantage of the spin flip method is substantial even if the polarized nature of the neutrons is not used.

A spectrometer can be constructed such that one has the option of using the spin flipper as monochromator or analyzer or both in place of the conventional crystal monochromator. In any case, a vertically bent PG crystal will be used to pre-mono-chromate the beam. Either a Be or PG filter is used to remove order contamination. The spectrometer will be capable of incident energies from 15 to 2 meV with resolutions from 1 meV to almost 10 μ.eV with adjustable resolution parameters. It should be noted that although the energy-dependent analyzer makes use of the neutron spin, the sample scattering need not be spin dependent.

## 3. Applications

### 3.1 Phonons

The triple-axis spectrometer was originally designed specifically for the purpose of measuring the phonons which a crystalline substance can sustain. A phonon is characterized by a quantized energy *ħω_s_*(***q***), momentum *ħ**q*** and eigenvector ***ϵ**_s_*(***q***). Here *s* numbers the various normal modes. This quasi-particle is used as a description of vibrations of the atoms and molecules which constitute the crystal. The energy *ħω_s_* is easily understood as the vibrational energy of the “normal mode,” the momentum *ħq*, relates the phase of the motions of the atoms whose vibrations constitute the phonon. Two atoms whose spatial position is determined only by the distance ***R***(*h*,*k*,*l*) between the crystalline unit cell in which they exist vibrate temporally with a phase difference of ***q****_s_* ·***R***(*h*,*k*,*l*), (*h*,*k*,*l*) being the cell indices of one cell with respect to the other. The eigenvector *ϵ_s_* of dimension 3*N*, *N* being the number of atoms in the unit cell of the crystal, describes in detail the spatial character of the vibrational mode. One consequence of such a description of the mechanical vibrations of the atoms and molecules is that *ω_s_*, and *ϵ_s_* are functions of ***q***. The detailed dependence is governed by the interactions between the atoms and molecules of the crystal. An important reason for the experimental determination of the values of *ω_s_*(***q***) as functions of ***q*** (termed the dispersion curves) is the investigation of these interactions. A neutron when scattered by a substance can interact with these phonons if the requirements of energy and momentum are satisfied. That is, the neutron energy and momentum change must equal the phonon energy and momentum if a scattering resonance is to occur. This requires that

$$E-E_{0}=\pm \hbar \omega_{si}\left(q\right),$$ (8)

and

ℏQ=ℏ(q+τ), (9)

**Fig. 4:**
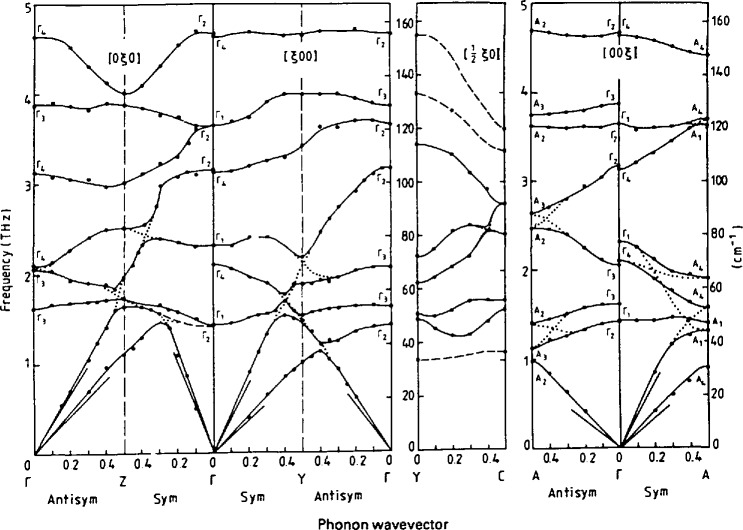
Measured dispersion curves for the 12 external and the 4 lowest internal modes in anthraene al 12 K for the [ζ00], [0ζ0] and [00ζ] directions. The presentation is given in the expended zone scheme such that branches must not cross. Some dispersion curves in the [0.5, ζ, 0] direction are also shown [6].

*ħτ* being a reciprocal lattice vector of the crystal [2]. The fact that these two quantities can be readily controlled by the triple axis spectrometer makes it the obvious choice as the instrument for their determination. Many substances have yielded to such measurements. The more complicated the unit cell (in number of atoms or molecules), the more complicated is the pattern of dispersion curves. An example of a rather complete measurement of the dispersion curves of a moderately complex crystal is presented in Fig. 4. The crystal is of deuterated anthracene [6] (C_14_D_10_). There are two molecules in the primitive cell giving rise to 12 external modes, 3 translational and 3 rotational for each molecule. There are symmetry-imposed requirements on the properties of the phonons. For those values of ***q*** for which these restrictions are greatest, a consequence is that substantial degeneracy occurs in the values of the eigenfre-quencies of several normal modes thus restricting the number of independent oscillators to be measured. This is reflected in the data presented in Fig. 4. Even with these restrictions, there still remain a substantial number of energy levels *ħω_s_*(***q***) for each ***q***. In practice an approximate knowledge of the eigenvector *ϵ_s_*,(***q***) is extremely useful in planning a strategy for the measurement and increasing confidence in the correct identification of the observed resonances. Equation (51) of Berk’s [2] article gives the cross-section dependence on these quantities. These eigenvectors are usually obtained from a first approximation of a model describing the crystalline interaction. There are also model independent group theoretical sum rules [7] for the structure factor which have proved very useful in the past [8].

### 3.2 Rotational Diffusion

**Fig. 6:**
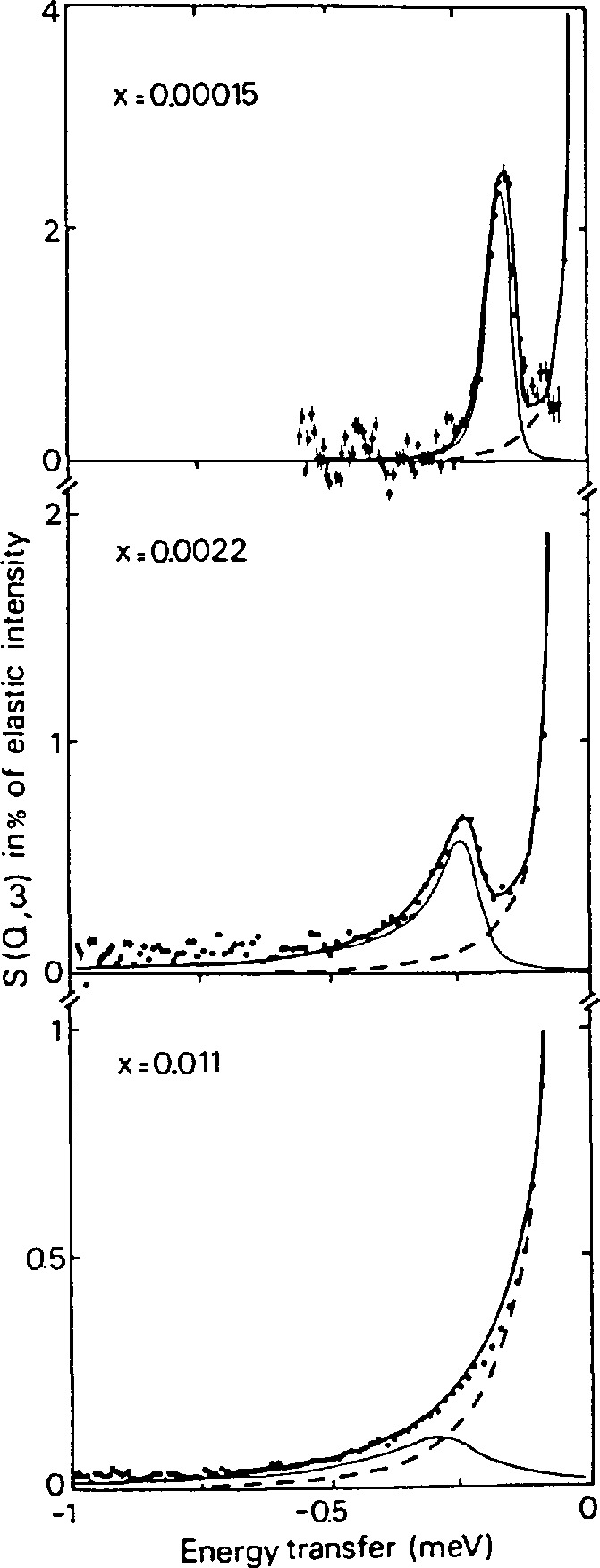
Tunneling spectrum measured at three concentrations for Nb(OH)*_y_* at 1.5 K (after Ref. [11]).

Diffusional motion of atoms whether they be local, such as rotational, or non-local, translational, are detectable in neutron scattering through the quasielastic spectrum which they produce. The energy width of the broadened line centered at *ħω* =0, and the *Q* dependence of its intensity with respect to the unbroadened elastic line contain information of both the structural and dynamical character of the motion [2]. The time scale of the motion available in neutron spectroscopy is inversely proportional to the energy resolution of the instrument, hence the motivation for CNRF triple axis spectrometer. The most extensively studied motions are those involving the hydrogen atoms both because it has the largest incoherent scattering cross section for neutrons and because its light mass produces motions of the appropriate time scale. Motions of other light atoms are not excluded and have indeed been investigated. The example used here is that of the rotational dynamics of the ammonia molecule in Ni(NH_3_)_6_I_2_ [8]. In the fcc phase of this material, the ammonia molecules are coordinated with the Ni such that the threefold axis of the molecule is directed toward it. This allows for rotation of the molecule about this threefold axis. In Fig. 6 is presented the temperature dependence of the quasielastic scattering spectrum from this substance. Several features are to be noted. The existence of both an elastic and quasielastic component is characteristic of localized diffusional motion. As the temperature increases, the energy width of the quasielastic increases reflecting a shortening of the rotational diffusion time of the molecule. At 5 K, this time is so long that the quasielastic peak is too narrow to be resolved, and at 120 K the time is so short, leading to such a broad quasielastic peak as to be almost unobservable. The temperature and ***Q*** dependence of the spectrum yields quantitative results for the structural model, residence times and thermal activation energy of this motion.

### 3.3 Incoherent Inelastic Scattering

These are studies of motions of hydrogen atoms in materials for reasons of cross section given above. Because of its light mass, hydrogen often finds itself in environments in which measurable quantum mechanical tunneling occurs. Many such situations occur for systems in which hydrogenous molecular groups (CH_3_, NH_3_, CH_4_,…) produce quantum tunneling in the presence of a reorientational potential [10]. We use here an example of H trapped at an interstitial oxygen impurity site in Nb(OH)*_y_* [11]. The tunneling spectrum of H is presented in Fig. 6 as a function of concentration. At very low concentrations there is only one narrow peak suggesting a small number of equivalent sites between which the H runnels. As the concentration increases, the tunneling peak broadens substantially. This has been interpreted as reflecting interaction between defects, even for these low concentrations, producing a distribution of local potentials. The properties of tunneling modes are extremely sensitive to the environment making their measurement a very useful probe.

### 3.4 Polarized Neutron Scattering

**Fig. 7:**
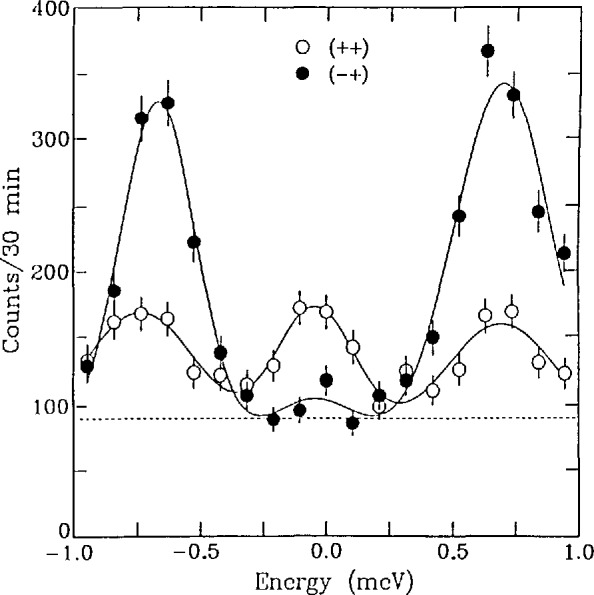
Observed scattering for *q*=0.009nm^−1^ (0.09Å^−1^) and 465 K in the vertical field configuration $\left(\hat{P}Q\right)$. The spin-flip scattering exhibits the usual spin wave excitations, while the non-spin-flip scattering also reveals longitudinal excitations near the spin wave energies (after Ref. [12]).

As mentioned above, the use of polarized neutron analysis of the scattering allows the separation of the scattering due to nuclear from that due to magnetic interactions. This powerful method of analysis and the fact that the neutron efficiently couples to the magnetic properties have resulted in very extensive applications of neutron scattering from magnetic materials. A most dramatic demonstration of the usefulness of this technique is represented by its use in detecting spin wave excitations of energy equal to longitudinal fluctuations of the atoms in amorphous ferromagnetic Fe_86_ B_14_ [12]. This material belongs to the Invar system of ferromagnets. Magnets belonging to this system of materials are exceptional as ferromagnets in that they seem not to conform to all the results of linear spin wave theory. An explanation of this behavior requires that there exist non-magnetic fluctuations which couple with the magnetic excitations. These “hidden” fluctuations should exist with very similar energy and form as the magnetic ones. Figure 7 presents a measurement of this effect. The scattering labeled (+ +) is measured with the magnetic fields arranged so that non spin flip neutrons are detected and labeled (− +) so that only those neutrons whose polarization direction is reversed by the scattering are detected. The spin flip is caused by magnetic scattering whereas the non spin flip by non-magnetic scattering by the nuclei. It is clear from these data that there exists excitations of both kinds at very similar energies. The authors strongly suggest this as having demonstrated the source of the “Invar anomaly.” It is also clear that only by polarized neutron scattering could such a result have been obtained.

## 4. The CNRF Instruments

**Fig. 8:**
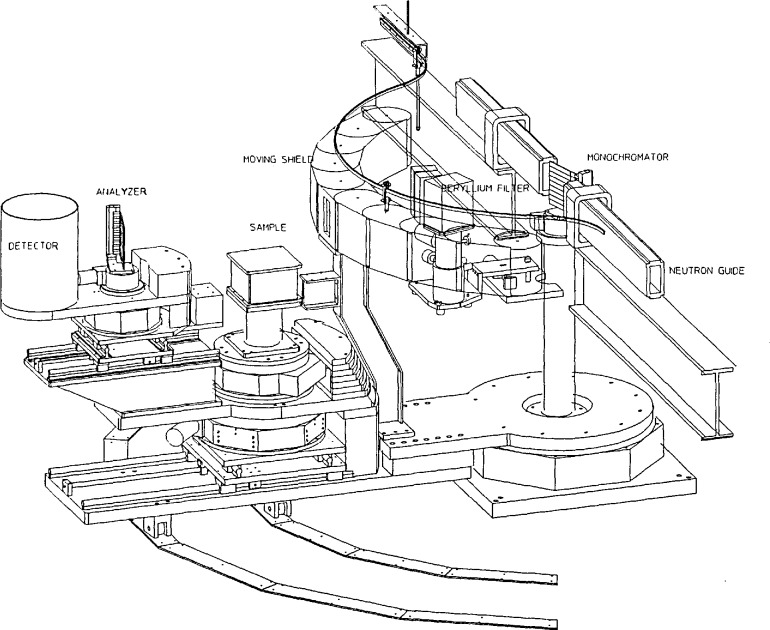
A schematic diagram of the CNRF triple axis spectrometer. The instrument is shown without the SPINS device.

Figure 8 presents a schematic diagram of the instruments under construction at NIST for installation on guide tubes at the CNRF. The configuration as presented includes the crystal analyzer which can easily be removed if the Drabkin flipper is used in its place. Both instruments will be constructed from the same design in order to minimize design and construction costs. Their function will depend on easily changed configurations. At present, the monochromator consists of vertically focused PG crystals followed by interchangeable (at the push of a button) PG filter, cooled Be filter, open hole or opaque beam stop. The collimator between monochromator and sample is carried within a movable shield attached to the monochromator to sample arm. This shield is constructed in the form of a “bicycle chain” which continuously covers the gap in the fixed monochromator shield. The position of the sample table is continuously variable along the line to the monochromator on accurate guides with a total travel of 670 mm. This feature allows the placement of polarizers, additional filters or energy-dependent filters before the sample. The analyzer table is also movable along the line to the sample allowing such devices to be placed between the sample and analyzer. The structure which carries the sample and analyzer tables is supported by two steel wheels rolling on flat steel plates. The analyzer and detector are supported on an arm which rotates about the sample and are counter balanced. This configuration was chosen to minimize the floor contact of the spectrometer. The analyzer crystal will consist either of a flat PG crystal or a horizontally focused PG crystal — the two being easily interchanged. The horizontally focused analyzer will provide energy focusing in those situations where the collimators on either side of the analyzer can be removed because of relaxed ***Q*** resolution. All of the motions of the spectrometer are produced with stepping motors which are in turn controlled by a computer. A versatile code allows the scheduling of the function of the spectrometer and the storage of the data. The computer which performs the real time control function is connected by a network to a larger, faster multipurpose computer. Data transfers between the two computers are readily accomplished. A substantial package of software exists on the larger computer for use in the analysis and reduction of the raw data.

## Figures and Tables

**Fig. 5:**
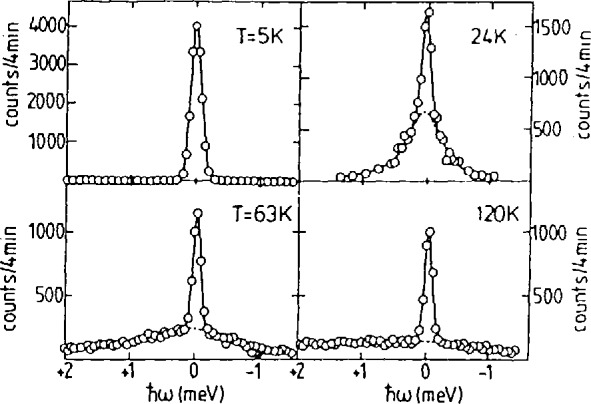
Temperature dependence of the quasielastic scattering spectrum from NI(NH_3_)_6_I_2_ (after Ref. [9]).

**Table 1:** Relative intensity *I* and energy resolution Δ*E* obtained with a single spin flipper and 80 min collimation compared with that obtained with a conventional triple-axis spectrometer using PG(002) crystals and various collimations

Spin flipper			Triple-Axis							
			80^4^		40^4^		20^4^		10^4^	
	Rel	Δ*E*	Rel	Δ*E*	Rel	Δ*E*	Rel	Δ*E*	Rel	Δ*E*
*M*	*I*	(meV)	*I*	(meV)	*I*	(meV)	*I*	(meV)	*I*	(meV)
50	1	0.320	1	0.308						
100	1/2	0.160			1/10	0.154				
200	1/4	0.080					1/33	0.077		
400	1/8	0.040							1/2000	0.039
